# Effects of sex steroids on thymic epithelium and thymocyte development

**DOI:** 10.3389/fimmu.2022.975858

**Published:** 2022-09-02

**Authors:** Matthew D. Taves, Jonathan D. Ashwell

**Affiliations:** ^1^ Laboratory of Immune Cell Biology, Center for Cancer Research, National Cancer Institute, National Institutes of Health, Bethesda, MD, United States; ^2^ Department of Neurobiology and Behavior, Cornell University, Ithaca, NY, United States

**Keywords:** thymocyte development, thymocyte selection, AIRE, androgens, estrogens, progestins

## Abstract

Sex steroid hormones have major effects on the thymus. Age-related increases in androgens and estrogens and pregnancy-induced increases in progestins all cause dramatic thymic atrophy. Atrophy can also be induced by treatment with exogenous sex steroids and reversed by ablation of endogenous sex steroids. Although these observations are frequently touted as evidence of steroid lymphotoxicity, they are often driven by steroid signaling in thymic epithelial cells (TEC), which are highly steroid responsive. Here, we outline the effects of sex steroids on the thymus and T cell development. We focus on studies that have examined steroid signaling *in vivo*, aiming to emphasize the actions of endogenous steroids which, *via* TEC, have remarkable programming effects on the TCR repertoire. Due to the dramatic effects of steroids on TEC, especially thymic involution, the direct effects of sex steroid signaling in thymocytes are less well understood. We outline studies that could be important in addressing these possibilities, and highlight suggestive findings of sex steroid generation within the thymus itself.

## Introduction

### The thymus

T cells are essential in the adaptive immune response to pathogens and tumors. Many core T cell programs and characteristics underlying their responses in the periphery are set during T cell development in the thymus. The thymus, therefore, is a key determiner of quantitative and qualitative characteristics of the adaptive immune response. The thymus is an encapsulated organ that is histologically divided into a cortex and medulla. Thymic epithelial cells (TECs), dendritic cells, and fibroblasts form a stroma through which developing T cells (thymocytes) migrate as they progress through various stages. The first step of T cell development involves the entry of circulating bone marrow-derived early T-cell progenitors (ETPs) into the thymus, *via* high endothelial venules. This entry occurs at the corticomedullary junction, and is driven by signals that include CCL25 (1). Cortical TEC (cTEC)-expressed DLL4 and IL-7 then drive T lineage commitment and proliferation of CD4^-^8^-^ (double negative, or DN) thymocytes as they migrate outward to the subcapsular zone (2, 3). DN4 thymocytes that have undergone successful T cell receptor (TCR)-β selection upregulate CD4 and CD8 to become double positive (DP) thymocytes, rearrange TCRα, and migrate inward through the cortex. TCRs are tested against thymoproteasome- and cathepsin L-generated peptides, which have unique cleavage sites and maximize the survival (positive selection) of thymocytes with functional TCRs (4–7). TCR signaling in cortical DP thymocytes upregulates TCR and CCR7 expression and trafficking to the thymic medulla. This coincides with the phenotypic transition to the CD4^+^CD8^-^ or CD4^-^CD8^+^ (single positive) phenotype. In the medulla, medullary thymic epithelial cells (mTEC) express antigens that are otherwise only found in peripheral tissues (i.e., tissue-restricted antigens, or TRAs), which are cross-presented by dendritic cells and test TCRs against a broad array of self antigens. Strongly self-reactive thymocytes that express high levels of TCR-induced proteins such as Nur77 (8, 9) undergo negative selection (death) or strong agonist selection (i.e., diversion into alternate lineages, such as Treg) (10, 11), establishing central tolerance and prevention of autoimmunity. TRA expression is driven by the transcriptional regulators Aire and Fezf2, which stochastically drive ectopic expression of thousands of genes in mTEC expressing high levels of MHC class II (mTEC^hi^) (12–14).

### Steroids

**Figure 1:**
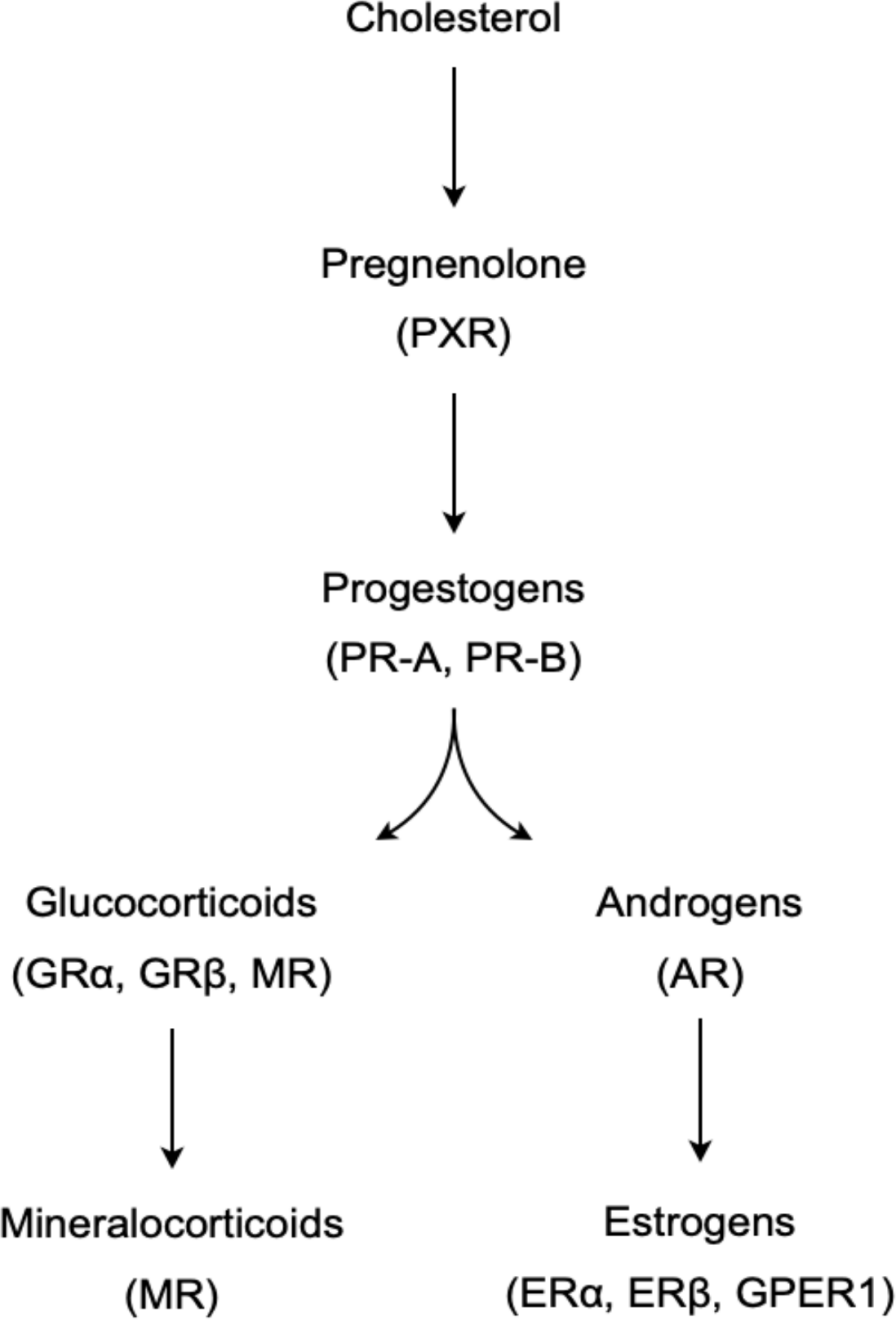
A simplified model of the steroidogenic pathway, showing the synthetic pathway of major steroid groups and their primary receptors in parentheses.

Gonadal secretions, later identified as steroids, were among the earliest classes of signaling molecules that were recognized to have potent effects on the thymus (15–17). Steroids are small, lipophilic hormones derived from cholesterol *via* the stepwise action of a cascade of steroidogenic enzymes (**Figure 1**) (18). The particular suite of available and active enzymes determines the steroid products generated by a given tissue, with sex steroids (estrogens and progestins in females, androgens in males) classically produced by the gonads (female ovaries and male testes), and corticosteroids (glucocorticoids, mineralocorticoids) classically produced in the adrenal cortex. Gonad- and adrenal-secreted steroids function as classic endocrine hormones, circulating systemically to coordinate organismal development or responses to various stimuli. Steroids act on target cells primarily *via* binding to nuclear receptors, which are ligand-activated transcription factors that regulate expression of large portions of the genome. Steroids were initially categorized into groups by their major actions in the body: androgens as ‘masculinizing’ agents, estrogens as ‘feminizing’ agents, progestins as gestation-promoting hormones, glucocorticoids as energy-mobilizing hormones, and mineralocorticoids as regulators of electrolyte balance. Because these activities are mediated by genetically and functionally distinct receptors, steroids are now broadly characterized by their primary intracellular receptors, the androgen receptor (AR), estrogen receptor (ER), progesterone receptor (PR), glucocorticoid receptor (GR), and mineralocorticoid receptor (MR) (19). Each of these steroid classes can also bind membrane-associated receptors (e.g. the G protein-coupled estrogen receptor, GPER1) to induce second messenger or protein kinase cascades that have rapid nongenomic actions on target cells (20). These are generally considered to be more minor activities.

The initial identification and isolation of steroids in the 1930s was followed by studies examining their physiological actions in animals, especially when given in large doses. One of the most distinct and consistent results was the response of the thymus to progestins, androgens, estrogens, and glucocorticoids. Large doses of these steroids resulted in a rapid and dramatic reduction in thymus size (21–23); the degree of thymus involution was even used as a bioassay to quantify steroid samples of unknown concentrations (24). This led to the notion of steroids as directly lymphotoxic molecules, which was supported by observations that endogenous sex steroid increases during puberty and pregnancy corresponded with thymic involution.

Steroids are central drivers of sexual development, and the types and concentrations of steroids in the blood are very different in females and males. These differences correspond with striking differences in immunity. Women compose the overwhelming majority of patients with autoimmune disease (>80%) (25) and are at greater risk of immune response-related pathology, whereas males are more susceptible to cancer and infection (26). This has long been attributed to the immunosuppressive actions of androgens and immunostimulatory actions of estrogens in peripheral immune responses. However, the dramatic responsiveness of thymi to steroids also raised the possibility that sex steroids might differentially affect the female and male thymus. In addition to differences in overall thymic output (27), recent data suggest there are important differences in the T cell repertoire. Given that sex steroid receptors are widely and variably expressed in thymic cell subsets (**Table 1**), it not surprising that steroids would have pleiotropic effects on the thymus and T cell development. In this minireview we provide an overview of the ways in which sex steroids regulate thymocyte development and the resulting composition of the peripheral T cell compartment.

**Table 1:** Steroid receptor gene expression across different thymus cell subsets.

Steroid receptor gene (protein)	Expression level					
	DN	DP	CD4	CD8	cTEC	mTEC
*Pgr* (PR)	+	+	+	+	+++	++
*Nr1i2* (PXR)	+	+	+	+	+	+
*Ar* (AR)	+	+	+	+	+++	++
*Esr1* (ERα)	++	+	+	+	++	++
*Esr2* (ERβ)	+	+	+	+	++	+
*Gper1* (GPR30)	+	+	+	+	+++	++
*Nr3c1* (GR)	++	+++	++	++	+++	++
*Nr3c2* (MR)	+	+	+	+	+++	++

Gene expression of steroid receptors across major thymus cell subsets. Data are compiled from multiple sources including the Immunological Genome Project database. Relative expression across subsets is normalized individually for each gene (i.e. “+” for two different genes does not indicate equivalent expression.

## Androgens

Androgens have historically been defined as steroids that stimulate the development of male characteristics, and are predominately produced by the testes. In males and females, however, androgens, especially inactive androgen precursors such as dehydroepiandrosterone (DHEA), are also produced by the adrenals and, in limited quantities, by the ovaries. Androgens act primarily by binding the AR, although androgen activation of membrane-associated receptors has been reported (28). In the periphery, androgens are generally thought of as being immunosuppressive, due in large part to suppression of cytokine production and cytotoxic effector function of activated T cells (29, 30). Furthermore, conditional deletion of AR in Treg cells reduces their number and increases Th2 cell numbers in allergic lung responses, implicating androgens as important suppressors of allergic responses in females and males (31). However, as detailed below, the immunosuppressive effects of androgens *in vivo* are likely largely due to their effects on thymocyte development and selection.

### Thymic involution

Exogenous and endogenous androgens have potent effects on the thymus, with castration or inhibition of androgen synthesis causing dramatic thymic enlargement, and treatment with androgens causing atrophy (17, 21, 32). This is driven by signaling through the classical AR, since AR-deficient (ARKO) male mice have thymi that are twice the size of wild-type controls (33, 34) and are completely refractory to treatment with exogenous androgens (33, 35).

Androgen treatment *in vitro* causes DP thymocyte apoptosis; this is largely mediated through induction of DP TNFα production and is blocked by addition of anti-TNF antibodies or knockout of *Tnf* (36). However, this does not seem to be a major contributor to androgen-induced thymic atrophy *in vivo*. Rather, experiments using radiation bone marrow chimeras found that it is AR expressed by stromal cells, not thymocytes, that mediate the effect of androgens on thymus size (33, 35). These findings were confirmed using AR conditional knockout mice, in which the thymus was found to be of normal size in thymocyte ARKO and fibroblast ARKO mice, but much larger in TEC ARKO mice (33). It is the thymic epithelial compartment, therefore, that drives androgen-induced thymic involution. Of note, TEC ARKO thymi were not as large as global ARKO thymi, which may have been due to poor deletion of AR in cortical cTEC (34) or, perhaps, to contributions by another cell type. Androgen signaling in TEC appears to mediate changes in thymus size by inhibiting TEC proliferation (37) and inhibiting TEC expression of molecules that promote thymocyte survival and proliferation, such as *Ccl21* and *Il7* (33, 38).

### Cortex

Androgens inhibit thymic seeding with ETPs, as there are fewer ETPs in castrated male mice (39). This was not due to effects on bone marrow hematopoiesis, as injection of T cell-depleted congenic bone marrow into castrated male mice resulted in increased numbers of DN thymocytes and later increased numbers of DP thymocytes (39). This was due to androgen inhibition of CCL25 expression by TEC, especially mTEC (38, 39). Androgens also suppress TEC expression of DLL4 (a Notch ligand critical for T lineage commitment) and IL-7 (which promotes DN survival and proliferation) (38), and in the case of the *Dll4* but not *Ccl25* and *Il7* the presence of androgen receptor binding sites in the promoter region. Chromatin immunoprecipitation and reporter plasmid experiments with the *Dll4* promoter demonstrated that binding of the liganded AR to androgen response elements was sufficient to increase gene expression (38). Finally, chemical inhibition of androgen production increased cTEC *Dll4* expression and thymocyte expression of Notch target genes, aand resulted in maximal expansion of DN, DP, CD4+CD8-, CD4+CD8- thymocyte numbers by 1, 2, 2, and 4 weeks, respectively (38). Interestingly, castration of RAG1-deficient mice causes a dramatic increase in the numbers of DN cells but not cTECs (37), suggesting that the inhibitory roles of androgens on early thymopoiesis are mediated largely by effects on cTEC-expressed molecules and not cTEC proliferation or survival.

Investigation of TEC ARKO thymi found no change in DP thymocyte proliferation (measured by BrdU incorporation) but a reduction in DP apoptosis (proportion of Annexin V^+^ cells) an elevated proportions of DP CD69hi cells, an indication of TCR-mediated signaling by self antigens (33). Together, these findings suggested that the increased cellularity of TEC ARKO thymi is due to enhanced positive selection. This was specifically tested by generating TEC ARKO mice expressing the CD4-restricted AND TCR transgene, a model of positive selection. These mice had a much larger thymus and increased proportions of DP CD69^+^ and CD4^+^CD8^-^ cells, all consistent with enhanced positive selection (33). Female TEC ARKO mice expressing an H-Y-specific TCR, in which thymocytes are positively selected on MHC I, had a similar phenotype except that the results of selection led to increases in CD4^-^CD8^+^ cells (33). These results indicate that androgens, acting *via* as-yet unclear AR signaling pathways in TEC, inhibit antigen-specific thymocyte positive selection.

### Medulla

The remarkable disparity in autoimmunity between females and males led to the idea that negative selection in the medulla might be affected by sex steroids. Early studies found that mTEC^hi^ growth was especially sensitive to androgens and that they rapidly proliferated after castration (37). Subsequently a pair of intriguing studies showed that negative selection is in fact dramatically responsive to sex steroids. Comparison of female and male thymi found that expression of *Aire* and *Aire*-dependent TRAs to be higher in males than in females, both mouse and human (40, 41). Correspondingly, androgen treatment of human TEC *in vitro* or mice *in vivo* upregulated expression of *Aire* and *Aire*-dependent TRAs. Sex differences in *Aire* expression were lost when castrated males were compared with females. In cultured human cells, the liganded AR was found to bind the *Aire* promoter and directly upregulate its expression (41). Male mice are known to be less susceptible than females in many models of autoimmunity, including experimental autoimmune encephalitis (EAE) and experimental autoimmune thyroiditis (EAT). Remarkably, the induction and severity of EAE (41) and EAT (40) were the same in *Aire*-deficient male and female mice, demonstrating that, at least in these models, the sex difference in the predisposition to autoimmunity is entirely *Aire*-dependent (and therefore due to sex differences in Aire expression). Furthermore, peripheral administration of the androgen dihydrotestosterone protected against EAE in control but not *Aire*-deficient mice (41). Therefore it is androgen signaling in the thymus, and not suppression of the peripheral immune response, that appears to be the primary driver of sex differences in autoimmunity.

## Estrogens

Estrogens are generally defined as steroids that regulate the development and activity of the female reproductive system and secondary sex characteristics. Estrogens, especially 17β-estradiol (estradiol), the most potent form, are primarily produced by the ovaries, although they can be produced in other tissues such as the brain (42). Estrogens signal *via* multiple receptors: the nuclear receptors ERα (*Esr1*) and ERβ (*Esr2*), and the membrane G-protein coupled receptor GPER1 (*Gper1*). In peripheral T cells, estrogens are primarily immuno-enhancing at low concentrations inducing T cell expression of T-bet and IFNγ (43) to promote Th1 responses, and at higher concentrations inducing Gata3 and IL-4 to promote Th2 responses (44). This skewing toward Th1 *versus* Th2 is considered a central driver of the female bias in autoimmunity.

### Thymic involution

Both exogenous and endogenous estrogens affect thymus size, with oophorectomy or estrogen synthesis blockade resulting in thymic hypertrophy in females and estrogen treatment causing atrophy in females and males (17, 21, 45, 46). This is primarily due to signaling *via* ERα, as *Esr1* knockout mice are partially resistant to thymic involution caused by exogenous estrogen administration (47, 48). Male and female *Esr2* KO mice, on the other hand, are similar to wild-type in their response to administered estrogen (49). *Gper1* KO mice have an intermediate phenotype, with a moderate reduction in thymus size in response to exogenous estrogens (47). It appears, therefore, that ERα and GPER1 both contribute to regulating thymus size.

As with androgens, estrogen effects on thymus cellularity appear to occur predominantly by signaling *via* the stromal compartment. Radiation bone marrow chimera experiments have shown normal thymus size in male WT recipients reconstituted with *Esr1* KO bone marrow but dramatically reduced thymus size in male *Esr1* KO recipients receiving WT bone marrow (48). Complementary radiation bone marrow chimera experiments found that exogenous estradiol caused thymic involution by signaling in both the stromal and hematopoietic compartments, as *Esr1* KO recipients of WT bone marrow had greater reduction in thymus cellularity than Esr1 KO recipients of Esr1 KO bone marrow (48). Importantly, these chimera experiments were not performed in female mice, in which endogenous estrogens would presumably contribute, especially in the absence of exogenous estradiol.

### Cortex

Surprisingly, Esr1 KO male and female mice have reduced thymus size (47, 48), indicating that basal estrogens actually play a role in promoting normal thymus growth. In spite of this, exogenous estradiol inhibits early stage thymocyte development, with accumulation of DN1 thymocytes and depletion of DN2, DN3, and DP thymocytes (47). This is not mediated in the same way as by androgens, as estrogens have little or no effect on Notch signaling (50). Instead, ERα (but not ERβ or GPER1) reduces IκB phosphorylation in DN cells, promoting IκB sequestration and inhibition of NF-κB signaling (47). As NF-κB signaling promotes survival and proliferation of β-selected thymocytes, its inhibition by liganded ERα may contribute to developmental arrest at this checkpoint (50). Exogenous estradiol also increases the proportion of apoptotic DN TCRβ^low^ thymocytes, an effect that is lost in *Gper1* KO but not *Esr1* KO or *Esr2* KO mice (47). Consistent with this, activation of GPER1 by a selective estrogen agonist induced moderate thymic involution and thymocyte apoptosis, but did not lead to developmental block of DN cells (47). Therefore the nuclear and membrane estrogen receptors have distinct functions: signaling *via* ERα inhibits cortical DN development whereas signaling *via* GPER1 selectively promotes DN cell apoptosis.

### Medulla

As mentioned above, *Aire* and *Aire*-dependent TRA expression are higher in males than females, and estrogen treatment downregulates expression of Aire and Aire-dependent TRA genes (40, 41). Estrogen was shown to induce methylation of the *Aire* promoter and reduce its expression whereas dihydrotestosterone had no effect (40). Dose-response studies with human TEC found that the androgen:estrogen ratio determined whether *Aire* is up- or down-regulated, and at least a 10-fold higher concentration of androgens was required to overcome estradiol-mediated downregulation (40). To extend these findings *in vivo*, fragments from the same male or female human thymus were grafted to female and male mice and relative *AIRE* gene expression was quantified. At day 4 *AIRE* expression was similar in human thymus fragments grafted to either sex. However, at 20 days *AIRE* expression in the human thymus was much lower in female than male mice. Consistent with a more potent effect of estrogens, *AIRE* expression in male recipients was similar at 4 and at 20 days, but *AIRE* expression in female recipients dropped dramatically from 4 to 20 days (40). These data show that androgen and estrogen signaling antagonize each other, with directly opposing effects on *Aire* expression and activity. Differences in antigen presentation may be further exacerbated by the fact that estrogen signaling reduces TEC expression of MHC (51).

**Figure 2:**
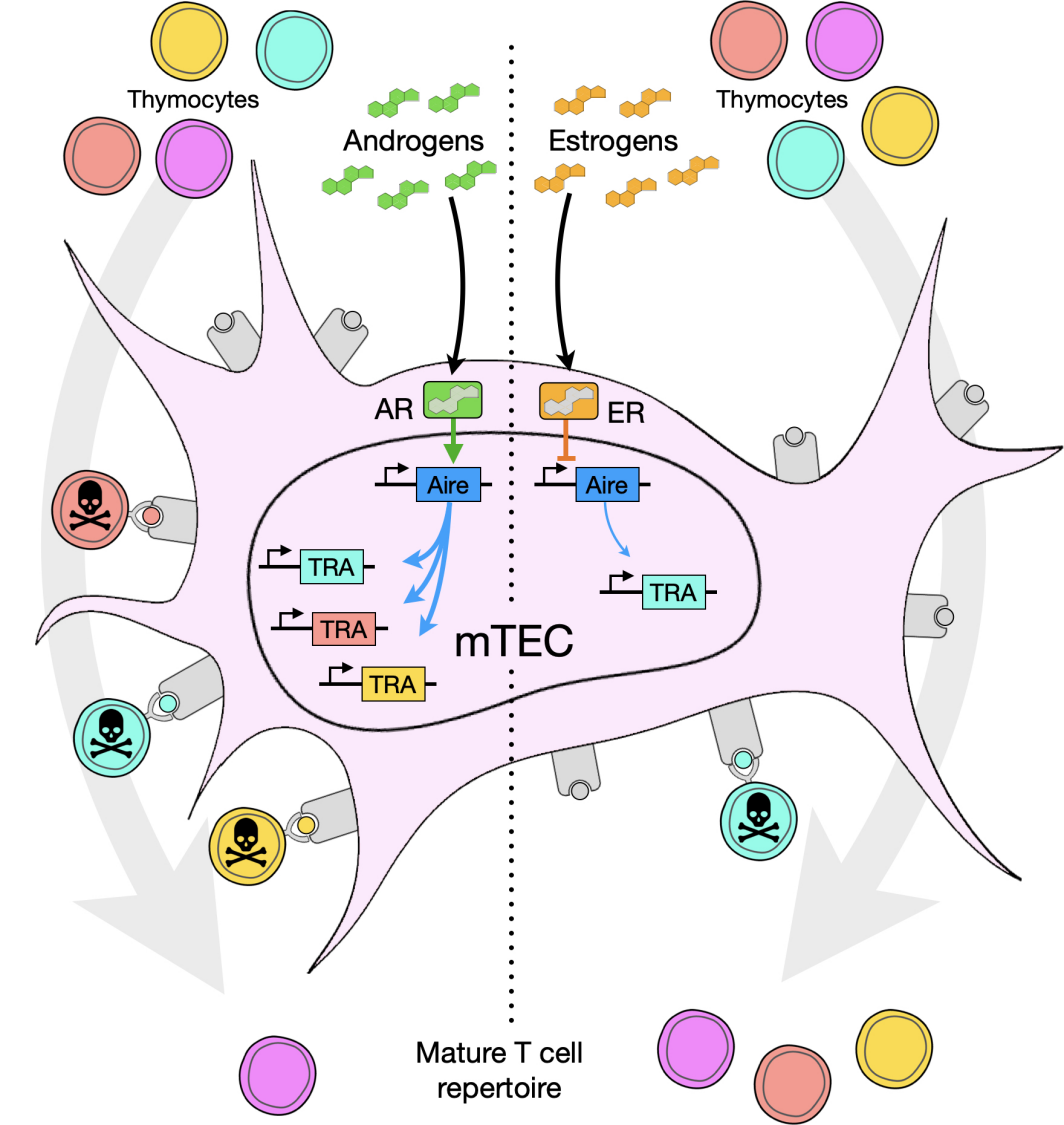
Model of steroid effects in the thymic medulla, especially on medullary thymic epithelial cells (mTEC). Androgens or estrogens bind the androgen receptor (AR) or estrogen receptor (ER), respectively, and up- or down-regulate expression of *Aire*. Aire in turn promotes, to a greater or lesser degree, expression of tissue restricted antigen (TRA) genes, generating an array of self-peptides presented on the surface of the cell bound to MHC molecules. Thymocytes with TCRs recognizing TRAs (shown as color-matched TRA, peptide antigen, and thymocyte) undergo negative selection and are absent from the mature TCR repertoire.

Estrogen treatment increases disease severity in EAT, which has been attributed to signaling in peripheral T cells, in particular ERα- and ERβ-mediated induction of Th1 and Th17 responses *via* upregulation of *Tbx21* (encoding T-bet), *Rorc* (encoding RORγt), *Il17*, and *Il21* (52, 53). These in turn are proposed to drive increases in autoantibody production (52). However, thymectomy abolished the disease-enhancing effect of estrogens without affecting autoantibody titers (40). To test the specific contribution of *Aire* to EAT severity, 7-week-old male mice received intrathymic injections of recombinant adeno-associated virus (AAV) miRNA to knock down endogenous *Aire* expression. Anti-*Aire* miRNA treatment reduced *Aire* transcript abundance in the male thymus by approximately 80% compared to control miRNA, which resulted in EAT pathology similar to that in females as quantified by autoantibody titers and numbers of thyroid-infiltrating CD8^+^ T cells (40). Together, these data indicate that inhibition of *Aire* expression and medullary negative selection, at least in this model, is a primary mechanism of estrogen-induced immunoenhancement. An overview of androgen *versus* estrogen effects on *Aire* and thymocyte selection is shown in **Figure 2**.

## Progestins

Progestins, in particular progesterone, are defined as steroids that support gestation. Progestins are primarily generated in the ovary and to a lesser extent in the adrenals, brain, and adipose tissue (18). During pregnancy the placenta is a major source of progesterone. Progesterone acts primarily by binding the intracellular PR, but also signals *via* membrane PRs and the cytosolic pregnane X receptor (PXR). In the periphery, progestins are well known to be immunosuppressive, antagonizing TCR signaling, suppressing expression of proinflammatory cytokines, and promoting Treg differentiation (54–56). Treg differentiation in particular appears to be a critical tolerogenic mechanism in pregnancy, with PR-deficient mice having dramatically lower maternal immune tolerance to the fetus (57).

### Thymic involution

Exogenous (21, 58) progesterone potently induces thymic involution, as does the elevated level of endogenous progesterone during pregnancy (58–60). Unlike androgens and estrogens, however, progestins do not seem to be a major contributor to age-related involution. Pregnancy-driven involution is mediated *via* signaling through the canonical intracellular PR (gene name *Pgr*), as thymi of *Pgr*-deficient mice are refractory to pregnancy-induced involution (58). Within the thymus, studies of radiation bone marrow chimeras have shown that stromal cells, rather than thymocytes, are the primary target of progesterone in driving involution (58). Very little pregnancy-induced thymic involution occurs in mice with TEC-specific *Pgr* KO, confirming that involution is almost completely mediated by PR signaling in TEC (59). Involution is global, with little or no change in the frequencies of different thymocyte subsets (59).

### Cortex

Progesterone reduces homing of ETPs to the thymus (60), which appears to be at least in part due to reduction in the homing chemokine CCL25 produced by epithelial cells (60). Elevated progesterone in pregnancy downregulates *DLL4* expression (60), reducing Notch signaling and T lineage commitment of the few ETPs that do enter the thymus. Once committed, however, subsequent thymocyte developmental progresses without any major blocks (58, 59).

### Medulla

Pregnancy is a unique immunological context, and maintenance of pregnancy is dependent on an appropriately selected TCR repertoire and sufficient induction of thymus- and peripheral-derived Treg cells (61). Interestingly, PR expression in the non-hematopoietic thymic compartment is necessary for normal fertility, as determined by the number of viable *versus* unimplanted and resorbed embryos (58). A few experiments have found that progesterone, like estradiol, reduces mTEC expression of *Aire in vitro* (40, 41), raising the possibility that PR-mediated decreases in *Aire* expression might reduce negative selection. However, a recent study reported that *Aire* and *Fezf2* expression were actually increased during pregnancy, and that this increase was lost in mice with TEC-specific *Pgr* KO (59). *In vivo*, therefore, it appears that progesterone signals through PR to increase *Aire* expression, presumably enhancing negative selection and possibly even promoting agonist selection of Treg cells to result in immunological tolerance of the fetus. Whether progesterone regulation of *Aire* plays any role in fetal tolerance remains to be tested. An additional possible contributor to fetal tolerance (and the TCR repertoire in non-pregnant female and male mice) is the role of PR signaling within thymocytes themselves. Careful *in vitro* experiments using PR-deficient and GR-deficient thymocytes have shown that progesterone binds and activates the thymocyte GR (62). GR signaling is known to antagonize TCR signaling in thymocyte negative selection by opposing *Nur77* and *Helios* expression (63), and progesterone appears to antagonize thymocyte TCR signaling in a very similar way (62). Paired with high levels of progesterone within the thymus (64, 65), these data raise the intriguing possibility that progesterone can regulate antigen-mediated selection by inhibition of *Aire* expression and by antagonism of thymocyte TCR signaling. However, experiments will be necessary to specifically test each of these possibilities and their biological relevance *in vivo*.

## Future directions and conclusions

The studies described above have clearly identified effects of sex steroids on thymus involution, TEC gene expression and proliferation, and thymocyte survival and apoptosis (**Table 2**). However, there are many important questions that remain. For example, AR directly upregulates *Tcf7* (TCF-1) (29, 30), which promotes DN thymocyte expression of *RAG* genes and TCR components as well as DP thymocyte survival. This suggests that androgens may act directly on thymocytes at early stages of commitment and differentiation. Sex steroid receptors in thymocytes may also play a role TCR signaling and thymocyte selection, although this also is largely unknown. Both AR and ERs have been shown to interact with and inhibit Nur77 (66, 67), which is induced by TCR signaling in thymocytes and promotes negative selection (8, 9). Similar interaction between the GR and Nur77 contributes to mutual antagonism between glucocorticoids and TCR signaling (63), and raises the possibility that in addition to altering *Aire* expression in mTEC, androgens and estrogens might also antagonize biological responses downstream of TCR signaling. As with glucocorticoid signaling, this could result in an altered TCR repertoire without obvious differences in thymocyte numbers. Indeed, the altered selection of the AND and H-Y transgenic TCRs in the absence of sex steroid signaling (33) is consistent with such an effect. Another interesting possibility is that of sex steroid production directly within the thymus. Thymic epithelial cells can synthesize glucocorticoids *de novo* from cholesterol (68–70), and much of the enzymatic machinery that functions in glucocorticoid synthesis also functions in synthesis of progesterone and androgen and estrogen precursors (18). Together with the finding that the thymus has locally elevated concentrations of progesterone (64, 65), this raises the intriguing possibility of sex steroid production by the thymus itself. If so, this might suggest a role for paracrine sex steroid signaling within the thymus.

**Table 2:** Overview of steroid effects on thymus cells.

Steroid class	Cell type	Action
	cTEC	↓ CCL25, DLL4
Progestins	mTEC	↑ *Aire* & TRA expression
	thymocytes	↓ ETP homing ↓ negative selection?
	cTEC	↓ CCL25, DLL4, IL-7 expression
Androgens	mTEC	↑ *Aire* & TRA expression
	thymocytes	↓ ETP homing, T lineage commitment, positive selection ↑ negative selection
	cTEC	?
Estrogens	mTEC	↓ *Aire* & TRA expression
	thymocytes	↓ NF-κB signaling & DN development, ↑ DN apoptosis ↑ negative selection
	cTEC	?
Glucocorticoids	mTEC	?
	thymocyte	↓ TCR signaling & negative selection
	cTEC	?
Mineralocorticoids	mTEC	?
	thymocytes	?

↓, decrease; ↑, increase; ?, unknown.

## Author contributions

All authors listed have made a substantial, direct, and intellectual contribution to the work and approved it for publication.

## Funding

This work was supported by the Intramural Research Program of the Center for Cancer Research, National Cancer Institute, National Institutes of Health.

## Conflict of interest

The authors declare that the research was conducted in the absence of any commercial or financial relationships that could be construed as a potential conflict of interest.

## Publisher’s note

All claims expressed in this article are solely those of the authors and do not necessarily represent those of their affiliated organizations, or those of the publisher, the editors and the reviewers. Any product that may be evaluated in this article, or claim that may be made by its manufacturer, is not guaranteed or endorsed by the publisher.
